# Impacts of computer-assisted diagnostic assessment on sustainability of L2 learners’ collaborative writing improvement and their engagement modes

**DOI:** 10.1186/s40862-022-00139-4

**Published:** 2022-04-18

**Authors:** Natasha Pourdana

**Affiliations:** Department of ELT, Karaj Branch, Islamic Azad University, Karaj, Iran

**Keywords:** Collaborative writing, Computer-assisted, DIA, Higher-level skills, Learner engagement, Lower-level skills

## Abstract

Diagnostic assessment (DIA) is under-researched in second/foreign language education despite its common practice across a wide range of professions such as medicine, mechanics, and information technology. This study aimed at exploring the impact of computer-assisted DIA on the sustainable improvement of English-as-a-Foreign-Language (EFL) learners’ collaborative writing and engagement modes. To do so, 36 selected EFL university students were paired to collaborate on writing data commentary tasks whose performances were subjected to the teacher’s regular DIA in Google Meet and task-wise student reflective logs over ten weeks. Repeated-measures ANOVA indicated dyads’ significant progress in lower-level writing skills (sentence structure, word choice & grammar, mechanics), but no considerable progress in higher-level writing skills (organization and development). The results of one-way ANOVA showed the DIA impact on individuals’ sustainable writing improvement from the pretest to the immediate and delayed posttests. Students’ reflective logs were analyzed to explore their behavioral, emotional, and cognitive modes of engagement in computer-assisted DIA. Theme frequency analysis indicated the participants’ active behavioral engagement in terms of their notable amount of time per task. They conveyed their emotional engagement in the user-friendliness of Google Meet, real-time social presence, and low anxiety experienced with DIA. Their cognitive engagement was depicted by their major approval of DIA and positive self-assessment of writing improvement. Yet, some participants were critical to the prioritization of language form(s) over the content in teacher DIA practice. This study yielded pedagogical implications for the L2 teachers to blend DIA, task-based academic writing, and student collaboration in e-learning contexts.

## Introduction

Vygotsky’s (1987) social constructionist approach to the second/foreign language (L2) teaching and learning recommends the L2 teachers to “scaffold the L2 learners when they are working on a complex task, and mediate them individually to accomplish the task until their learning needs are fulfilled” (Rafi et al., 2022, p. 2). The L2 teacher individualized attention to the struggling L2 learners to obtain the fine-grained diagnostic information about them has become a dominant discourse in learner-centered pedagogy (Weimer, 2002) and assessment *for* learning (AfL) (Carless & Boud, 2018) that can be carried out in several educational trajectories including collaborative learning (Lei & Medwell, 2021), formative assessment (Lee, 2015), differentiated instructions (Pourdana & Rad, 2017), and diagnostic assessment (Tomlinson, 2014; Yin et al., 2012).

The paradigm shift of assessment from teacher-centered information-oriented to the learner-centered process-oriented approach might be accomplished in the classroom practice of diagnostic assessment (hereafter, DIA) (Alderson, 2005; Alderson et al., 2015; Jang & Wagner, 2014). In an ideal educational context, DIA provides L2 learners with detailed, individualized, and explicit information that can help them reflect upon their learning problems to take remedial actions. To benefit most from DIA, L2 learners also need to actively *engage* in appreciating feedback, making evaluative judgments, managing their negative affect, and taking action as the uptake of feedback information. The learner may use this information to “confirm, add to, overwrite, tune, or restructure information in memory, whether that information is domain knowledge, metacognitive knowledge, beliefs about self and tasks, or cognitive tactics and strategies” (Butler & Winne, 1995, p. 275). L2 learners' adopted agency and active engagement with feedback are conceptualized as *student feedback literacy* (Carless & Boud, 2018; Sutton, 2012) which can be maximized through the regular implementation of DIA by the L2 teachers (Chong, 2020). According to Carless and Boud (2018), feedback literacy, or the L2 learners’ “ability to read, interpret and use written feedback” (p. 2) is empowered and expanded when the teachers switch from traditional output-oriented assessment methods to launching observation, participation, and dialogue in DIA practice.

The second/foreign language acquisition (SLA) researchers' interest in DIA has recently been provoked by surging demands for tailor-made assessment alternatives that could address the sources of L2 learners’ learning problems and provide the L2 teachers with effective and accessible means to deal with nature and the root causes of their students’ cognitive strengths and weaknesses. Provided that DIA is the identification of the nature and causes of L2 learners’ problems, it should be an integral part of the language curriculum or at least every L2 teaching process. However, the pedagogical role of DIA in the field of L2 education has remained untested and teachers are rarely aware of *how* to diagnose their learners’ strengths and weaknesses (Harding et al., 2015). There is also limited empirical research on how teachers can diagnose L2 learners’ challenges when they *collaborate* in writing tasks. More specifically, the opportunity to integrate DIA in teaching academic writing skills in L2 educational contexts is still under-documented (Alderson et al., 2015; Cumming, 2015; Martin & Miller, 2003). By the same token, a few SLA studies have examined the delivery of DIA in the computer-mediated communication (CMC) online platform in the absence of the face-to-face modality of interaction, for instance, in distance learning or as a response to distressing situations such as the COVID-19 pandemic. Similarly, the SLA research on how DIA demands L2 learners to actively engage in generating sustainable uptake of DIA is still inconclusive (Gorin, 2007).

Therefore, the integration of DIA and collaborative academic writing on the virtual CMC platform seems an untaken road this study attempted to explore. The researcher’s objective was to examine the impact of DIA on (1) the short-term and long-term learning improvement of various writing skills (higher-level and lower-level) when the EFL learners collaborate on academic writing tasks and on (2) their behavioral, emotional, and cognitive modes of *engagement* in DIA as the major step to sustain their feedback uptake. The target audience in this study is the EFL practitioners, SLA, and assessment researchers.

## Literature review

### Diagnostic assessment in theory and practice

*Diagnosis* is defined as the “investigation or analysis of the cause or nature of a condition, situation, or problem” (Diagnosis 2013). It is often known as the responsibility of the L2 teachers to diagnose their students’ learning problems by constantly observing them, encouraging their learning efforts, and identifying the obstacles to their future learning progress (Alderson et al., 2015; Rafi et al., 2022). In this sense, DIA is similar to formative assessment as both involve the provision of ongoing feedback to the students so that its impact could affect their subsequent learning experience. However, while in the formative assessment the learners’ agency in the feedback uptake and the personalized use of feedback is limited, by receiving DIA, “the L2 learners can self-generate internal feedback by monitoring their cognitive engagement with a task” (Jang & Wagner, 2014, p. 4). Moreover, DIA is often supplemented with compensatory instructions and supportive activities to eliminate obstacles and facilitate decision-making.

According to Alderson, “focusing on strengths will enable the identification of the level a learner has reached, and focusing on weaknesses or possible areas for improvement should lead to remediation or further instruction” (2005, p. 256). Alderson et al. (2015) elaborated on several attributes of DIA which make it different from formative assessment. Accordingly, DIA is founded on five principles:

- It is personalized, learner-centered, and descriptive,
- It makes an explicit focus on the *therapy* (i.e., remedy) in the future performance,
- It provides a detailed analysis of the problematic areas which need assistance,
- It includes more discrete-point items than integrative items,
- It generates detailed, immediate, and observable feedback to the learners in “four diagnostic stages of (1) listening/observation, (2) initial assessment, (3) use of tools, tests, expert help, and (4) decision-making” (Harding et al., 2015, p. 2).

Harding et al. (2015) elaborated on DIA operational stages. Accordingly, in diagnosing the L2 writing skills, for instance, the L2 teachers might implement the DIA stages by (1) monitoring the participants’ writing difficulties, (2) giving directions to the problematic areas by giving authentic examples of writing sketches, (3) providing immediate, individualized and detailed diagnostic feedback to resolve the participants’ errors located in the task outcomes, regularly suggesting learning plans, and praising the strong points in the students’ writing, and (4) making post-intervention decisions.

To capitalize on the pedagogical role of DIA, Jang and Wagner (2014) differentiated evaluative feedback or *feedback on knowing* from cognitive feedback or *feedback for knowing*. By promoting DIA as a type of cognitive feedback, they argued that cognitive feedback provides descriptive information about the cognitive processes and strategies that L2 learners have/to be used in solving challenging problems (e.g., self-monitoring, visualization, L2 form-function mapping). Cognitive feedback is often diagnostic, formative, and facilitative that *engages* L2 learners in their performance on the tasks and effective translation of the feedback into action (i.e., task uptake). It “signals a gap between the learner’s current level of performance and the desired level of performance or goal” (Jang & Wagner, 2014, p. 6). However, evaluative feedback usually focuses on students’ task outcomes (e.g., rates and grades) rather than adopting effective cognitive strategies for solving problems. It is usually summative, excessively detailed, and corrective by nature which provides only marginal guidance to individual learners about how to make progress in their subsequent learning goals (Pourdana et al., 2012; Sutton, 2012).

By the informed practice of DIA, the L2 teachers can (a) observe the consistency of the L2 learner’s understanding with instructional objectives, (b) add missing information to their existing knowledge, (c) change their misconceptions, (d) adjust their understandings by classified concepts, and (e) reorganize their incompatible knowledge with new learning (Jang & Wagner, 2014). In the condition of regular DIA, L2 learners can also develop self-generated cognitive feedback which is the result of ongoing monitoring of their learning progress. Such internal cognitive feedback is a catalyst for self-regulated learning (Butler & Winne, 1995; Doe, 2015; Pourdana et al., 2014). As a result, both the content of the DIA and the type of tasks upon which DIA is based play critical roles in advancing students’ self-regulation. Among the most engaging modalities of L2 pedagogical tasks is collaborative writing.

Through collaboration, L2 learners no longer need to rely exclusively on their L2 knowledge resources when performing a challenging writing task. In other words, they have substantive interaction in all stages of the writing process to receive assistance from the teacher and/or peers (Storch, 2013). From this perspective, collaborative writing entails both a process and a product. In collaborative writing, therefore, the immediacy of assistance and resources enables L2 learners to co-construct the target language knowledge more effectively. Such assistance can be in the form of negative feedback (correction), positive feedback (confirmation), or diagnostic feedback (remediation). They also share their decision-making power and equally feel responsible for the produced artifact.

Collaborative writing sustains students’ close attention to different aspects of language skills development, including the higher or macro-level and lower or micro-level of target-language discourse. In Cumming’s (2001) discussion of “text features” in L2 writing, through learning the lower-level skills of writing, the L2 learners gradually increase the syntactic and morphological complexity and accuracy of their written texts. At the higher level of discourse, the L2 learners are adept to incorporate “a hierarchy of related ideas at the beginning, end, or throughout a text specifically by using cohesive, functional-semantic, or various stylistic devices” (Cumming, 2001, p. 4). Supported by the L2 writing literature, skilled writers at lower and higher levels tend to use the recursive cognitive sub-processes such as monitoring and revising their texts or ideas more extensively and successfully than the unskilled writers (Behbahani et al., 2011; Cárcamo, 2020; Pourdana et al., 2021).

On the other hand, as Lam (2018) speculated, collaborative writing generates and regulates L2 learners' active engagement in the writing tasks. *Engagement* is commonly considered as “a type of motivation, expressed in a particular moment through active involvement in the learning process” (Bonner et al., 2022, p. 2). According to Fredricks and Eccles (2002), learner engagement has three major components of behavior, emotion, and cognition. By their definition, *behavioral engagement* involves the learners’ active participation in terms of self-regulating on-task behavior which likely causes successful academic outcomes. *Emotional engagement* entails the learners’ both positive and negative responses to the teacher feedback, classroom tasks, and interactions with classmates which can engineer their willingness to communicate. Finally, *cognitive engagement* is the learners’ constant cognitive investment that facilitates their openness to self-regulated learning (Fredricks et al., 2004). The integration of behavior, emotion, and cognition seems critical to language learning development since it can sustain a long-term commitment in L2 learners.

Recently, the SLA researchers have shown a growing interest in DIA practice in L2 educational contexts. For instance, Rafi et al. (2022) investigated the differential impacts of computer-mediated DIA on mixed-ability non-English major university students’ learning of English words pronunciation. In the Google Meet platform, 66 participants completed graded words pronunciation tasks for 10 weeks. They received DIA on their committed errors, while they were praised for the accuracy of their pronunciation, and recommended the strategies for self-improvement. The statistical results reported significant progress in the participants’ words pronunciation. The participants’ responses to the post-intervention interview depicted their positive attitude towards the immediacy and ease of the DIA in the Google Meet platform. Similarly, Nikmard and Tavassoli (2020) adopted an output-oriented approach to examine the impact of DIA on English major EFL learners’ selective and productive reading task outputs. In their experimental research, 60 participants were divided into treatment and control groups who completed both selective and productive reading tasks with/without DIA, respectively. The results indicated the significant improvement of the treatment group on both selective and productive tasks.

### Computer-assisted diagnostic assessment

Advanced information and communication technologies (ICT) and computer-mediated communication (CMC) have recently revolutionized teaching, learning, assessment, and sharing information in education. They have also lifted the face of language assessment for summative and diagnostic purposes. ICT and CMC have great potential to advance the efficiency of DIA and the associated decision-making by the L2 teachers and learners as the stakeholders (Alderson, 2005; Chong, 2020). Unlike the teacher feedback, computer-assisted DIA rarely causes boredom in the students. On the contrary, by providing automated feedback, or offering L2 learners simple and practical suggestions to revise errors, CMC interfaces can play an effective pedagogical role in L2 learning (Ritter, 2018). As Csapó and Molnár (2019) rightfully argued, systematic DIA can hardly be conducted regularly by the teachers through paper-based instruments due to several restrictions such as large class size and shortage of time. Therefore, the technology-based DIA might be one of the best options available to L2 teachers and learners.

Among CMC platforms, Google Meet (formerly known as *Hangouts*) is a cost-free multimedia communication service, which has been developed by Google. Google Meet allows up to 100 participants to join a live-streamed meeting and have simultaneous face-to-face interactions. They can speak, record, and share texts, photos, videos, and multimedia messages with one another anywhere with Internet access. From a pedagogical standpoint, Google Meet can properly generate an interactive learning environment with dynamic group sizes, such as whole class, small groups, or dyads (Hismanoglu & Hismanoglu, 2011). Google Meet is also available as a digital application to download, install, and register into Microsoft Windows, Android, and IOS mobile operating systems.

The L2 research carried out in the Google Meet platform has increased in the last two years as a result of distance education imposed by the surge of the COVID-19 pandemic (Al-Maroof et al., 2020; Purwanto & Tannady, 2020; Putra, 2021; Sette-de-Souza, 2020). The common ground in these research reports is that online platforms such as Google Meet or Moodle are the possible solution to facilitate the L2 learning process at individual and group levels. In Google Meet video conferencing, the observations indicate a sense of togetherness in the students which motivates them to have more interactive communication (Rafi et al., 2022). Moreover, the visual display of the teacher and the learners can provide greater opportunities for low-stress participation, undivided attention, better noticing of errors, and meaningful negotiation about language use (Baber, 2020; Storch, 2013). The user-friendliness and reported efficiency of Google Meet, therefore, turn it into a suitable option to embed the DIA practice and collaborative writing in this study. In this study, a quasi-experimental time-series design was adopted and the following research questions were raised:

**RQ1** Does the computer-assisted DIA affect EFL learners’ control of higher-level and lower-level writing skills differently in their collaborative writing performance?

**RQ2** Does the computer-assisted DIA affect the EFL learners’ writing improvement and its sustainability?

**RQ3** How does the computer-assisted DIA affect EFL learners’ engagement in behavioral, emotional, and cognitive modes?

## Method

### Participants

This study was in the mid-COVID-19 pandemic launched on a large university campus in Iran. A total of 41 Persian-speaking (their L1) EFL freshman students of non-English majors registered to participate in this study. The non-random convenience sampling was adopted (Best & Kahn, 2006) to select a large enough sample of informants. The criteria for participation included (1) general English proficiency level at the intermediate level or above, (2) writing ability at the onset of the study evaluated with a writing pretest, and (3) experience of academic writing in English. The participants’ English proficiency level was measured by administering the virtual version of the Oxford Placement Test (Version 1, 2001). Due to the restrictions imposed by the COVID-19 lockdown, we converted the 60 items of the OPT into the Google Forms survey software, for virtual participation.

After signing a consent form, the volunteers worked on the electronic version of OPT for 45 min. The candidates whose OPT scores determined their language proficiency at intermediate level (30–37, B1 in OPT ranking system, M = 34.33, SD = 0.91) made up the majority (N = 36). Therefore, the five outliers were eliminated. The main sample of participants made up 18 self-selected dyads to complete collaborative writing tasks for 10 consecutive weeks. The researcher in this study was a university professor majoring in teaching English as a foreign language (TEFL) with 15 years of professional experience. Three Ph.D. candidates majoring in TEFL assisted the researcher in co-rating the writing tasks and student log analysis. Table 1 summarizes the demographic information of the participants.

**Table 1:** Demographic attributes of the participants

N	Gender	f (%)	Age range	f (%)	Major	f (%)	Studying English (Y)	f (%)	OPT range	f (%)
3	Female	28 (68)	20–24	27 (64)	Management	12 (44)	13	28 (72)	30–33	12 (44)
6	Male	8 (32)	25–31	9 (36)	Economy	18 (32)	11	8 (28)	34–37	24 (56)
					Mathematics	4 (16)				
					Accounting	2 (8)				

f = frequency; Y = year

### Instruments

#### Rating rubric

West Virginia Department of Education (WVDE) writing rubric (2011) (“Appendix 1”) was used to rate the collaborative data commentary tasks, the pretest, and the immediate and delayed posttests. The WVDE rubric was also used as the reference for the analytical diagnosis of the dyads in collaborative tasks. The rubric consists of two higher-level components of writing *organization,* and *development,* as well as three lower-level components of *sentence structure, word choice & grammar,* and *mechanics*. It also has 6-band scores, ranging from 1 (minimal) to 6 (exemplary) writing quality criteria. The WVDE writing rubric is well-known for providing an analytical and reliable measurement of student writing performance in English based on a statistical report by NBCT Office of Assessment West Virginia Department of Education (2015). Our logic behind adopting this rubric was its user-friendliness, clarity of rubric indicators, and creditability. The WVDE writing rubric was distributed and introduced to the participants as “a learning road map” before the treatment began to facilitate their understanding of the diagnostic feedback points they would receive (Mahmoudi & Buğra, 2020).

#### Collaborative data commentary tasks

Data commentary is a content-focused writing task that requires the L2 learners to interpret the information demonstrated in a graphic prompt or a table and convert it into the written script (Wigglesworth & Storch, 2009). According to Storch (2013), when data commentary tasks are completed through collaboration, they are deemed to improve language learning. The current researchers’ academic objective for developing data commentary tasks was to enhance the non-English major students’ ability in reporting tables, figures, flowcharts, and other course-related writing tasks in English at the university level.

Ten data commentary tasks were developed by the researchers (cf. a sample in “Appendix 2”). The topics were pooled after a topic familiarity checklist was distributed among the participants to determine their common grounds. The selected topics had a wide range including education, computer science, physics, and the Noble Prize winners’ biographies. They were prompted with graphics, diagrams, photos, and videos to engage the participants as much as possible. The participants were required to begin collaborative writing on the topic in the virtual sessions but complete the task collaboratively at home via Google Meet to have enough time for (re)drafting. The dyads were asked to keep a record of the time they spent on writing and revising per task.

#### Writing pretest, the immediate and delayed posttests

The participants were required to individually complete a production-based writing task which was assigned as the pretest, the immediate, and delayed posttests. Accordingly, they were asked to write an essay of around 300 words. Participants spent no longer than 30 min on this task on average. The pre-writing input was a short video on *Life Planning – 4 Steps To Plan A Great Future* (youtube.com). This topic was selected out of the responses to the topic familiarity questionnaire. The purpose of conducting a pretest was to reassure no significant differences preexisted in the writing ability of the participants.

After 10 weeks of intervention, an immediate posttest was administered to measure the possible post-intervention improvement, followed by a delayed posttest to measure the sustainability of the detected writing improvement, if any. The pretest, the immediate and delayed posttests were co-rated by the researchers (N = 4) independently following the WVDE rubric. The inter-rater reliability was calculated for the three administered tests, Cronbach's α = 0.909, 0.817, and 0.811, respectively. The controversies were consulted case-wise to reach a full agreement.

#### Student reflective logs

Student reflective logs were the input for the qualitative data collected in this study. They were written by the dyads and posted to their profiles on Moodle learning management system (LMS) after they received the teacher DIA on every collaborative task output. They were required to respond to a set of pre-scripted prompts in their reflective logs.

The reflective logs had three sections based on the dyads’ responses to the three prompts about the students’ engagement. The first prompt which addressed the behavioral mode of engagement was *How much time did you spend on writing and revising Task #?* Based on Fredricks and Eccles’s (2002) model of engagement, the behavioral mode of engagement was operationally defined as the length of time per task every dyad spent on writing and revising weekly assignments. The second prompt in the reflective log addressed the emotional mode of engagement and required the participants to retrospect on their personal experience after receiving the DIA on every task they completed together. It was *How do you describe your personal experience of teacher feedback on your Task # output?* The third prompt addressed the cognitive mode of engagement. It required the participants to report on what they had learned from the DIA and their self-assessment of their writing weaknesses by responding to *What did you learn from the teacher feedback and how did you apply it to Task # output?*

The dyads were allowed (but not recommended) to write their logs in Persian to express their thoughts easily and fluently. The Persian logs were analyzed similarly to the English reflective logs. The analysis and theme extraction were done collaboratively by all researchers and reached a 91% agreement. The controversies were negotiated case by case to obtain a full consensus.

### Procedure

In a repeated-measures design, the experimental procedure of this study was carried out in 10 weeks. The 40-min intervention was a fraction of the regular 90-min sessions in the General English course for non-English major university students. The participants’ collaboration on writing tasks was the partial fulfillment of the course requirements. A week before the study began, the OPT was administered online as the placement test. After selecting 36 volunteers whose scores determined their language proficiency at the intermediate level, the participants logged in to Google Meet for attending an online tutoring session. The researcher introduced the participants to the WVDE writing rubric, data commentary task, and DIA procedure in 90 min.

The course began with the individual participant’s performance on the writing pretest. In the following 10 sessions, the time was split into 50 min of the regular instructions to cover the general English course materials, and 40 min of dyads’ collaboration on writing data commentary tasks. The dyads were required to complete the task at home and post it to their Moodle profiles to share with the researchers within 24 h. Every dyad’s performance on the data commentary task of the week was subjected to the DIA in the following session. To do so, the researchers hosted parallel diagnosis sessions and provided collective diagnostic feedback on the committed errors. The common errors with higher frequency were given priority to receive more time and detailed focus. The researchers provided the participants with individualized assignments and learning plans. Moreover, the well-written structures in every posted task output were labeled as “the Selection of the Session” by the researchers and shared with the class. After receiving DIA on their task output, the dyads prepared a reflective log by responding to the three fixed prompts, either in L2 or L1. The course was ended with the participants’ individual performance on the 30-min immediate writing posttest. After a four-week interval, the participants individually performed on a delayed writing posttest of a similar topic.

Every week, the four researchers collaboratively carried out adding up the feedback points on the higher-level (i.e., organization, and development) and lower-level (i.e., sentence structure, word choice & grammar, and mechanics) writing skills addressed in the WVDE writing rubric. The feedback scores were announced privately on the dyads’ Moodle profile. Therefore, every participant received ten feedback scores on the completed data commentary tasks, three scores on the pretest, the immediate and delayed posttests.

## Results

The quantitative data in this study were subjected to repeated measures analysis of variance (RM ANOVA), and one-way ANOVA. Three research questions that were raised in the study investigated the potential of computer-assisted DIA (1) to generate differential impacts on the improvement of higher-level and lower-level writing skills in collaborative writing tasks, (2) to make sustainable impacts on the learners’ writing achievement from the pretest to the immediate and delayed posttests, and (3) to affect the EFL learners’ behavioral, emotional, and cognitive modes of engagement in DIA on collaborative writing. Before analyzing the collected data on the first and second research questions, the normality distributions of the diagnostic feedback points, the pretest, the immediate and delayed posttests gain scores were examined.

As Table 2 shows, the ratios of skewness and kurtosis were lower than ± 2 which retained the normality of the data (Bryne, 2010). To address the first research question, the researchers initially conducted descriptive statistical analysis followed by the RM ANOVA. It should be noted that the observed decrease in feedback points on the dyads’ errors was interpreted as the students’ progress in their writing ability.

**Table 2:** Testing normality assumption

	N	Skewness			Kurtosis		
		Statistic	Std. error	Ratio	Statistic	Std. error	Ratio
Data commentary task							
1	36	− .336	.295	− 1.123	.376	.592	.635
2	36	.502	.295	1. 719	− .218	.592	− .368
3	36	.498	.295	1.688	.314	.592	.530
4	36	.552	.295	1.868	.964	.592	1.628
5	36	.452	.295	1.532	.964	.592	1.626
6	36	.331	.295	1.122	.150	.592	.253
7	36	.331	.295	1.122	.150	.592	.253
8	36	.029	.295	.098	− .713	.592	− 1.204
9	36	.060	.295	.203	− .345	.592	− .583
10	36	.030	.302	.101	− .787	.592	− 1.329
Test							
Pretest	36	.172	.295	.583	− .010	.592	− 0.16
Immediate posttest	36	− .329	.295	1.115	.713	.592	1.204
Delayed posttest	36	− .552	.295	− 1.868	.376	.592	.635

### Differential impacts of DIA on higher-level and lower-level writing skills

Table 3 summarizes the descriptive statistics conducted with the feedback points on the higher-level and lower-level components of writing in 10 collaborative writing tasks. As displayed in Table 3, the average DIA points on lower-level writing components were relatively larger than the feedback points on higher-level components in Task 1 to Task 10. Moreover, Table 3 indicates a rather slower pattern of reduction in the feedback points on the higher-level components than the feedback points on the lower-level components task-wise.

**Table 3:** Descriptive statistics: higher-level and lower-level skills in collaborative writing tasks

Task	Statistics	Higher-level skills		Lower-level skills			Total
		Organization	Development	Sentence structure	Word choice & grammar	Mechanics	
1	Mean	6.11	6.88	19.94	21.97	18.07	14.59
	Std. deviation	.89	.50	1.10	1.10	.40	.87
2	Mean	6.42	6.64	17.50	18.00	18.09	13.33
	std. deviation	1.94	.53	.90	.90	.42	.76
3	Mean	5.01	4.81	14.09	16.09	16.70	11.34
	Std. deviation	.91	.60	1.96	.88	1.54	1.03
4	Mean	4.90	4.67	12.32	15.32	14.30	10.30
	Std. deviation	1.50	.54	.98	.90	.44	.82
5	Mean	4.87	4.09	10.80	10.72	14.70	9.03
	Std. deviation	1.20	.40	1.53	.44	.52	.69
6	Mean	4.81	4.83	10.20	9.11	11.20	8.03
	Std. deviation	1.42	.43	1.40	.70	.90	.94
7	Mean	4.01	4.81	9.09	11.09	11.70	8.14
	Std. deviation	.91	.60	1.96	.88	1.54	1.03
8	Mean	4.90	4.67	11.32	11.72	8.30	8.18
	Std. deviation	1.50	.54	.98	.90	.44	.82
9	Mean	4.17	4.09	10.50	9.42	8.70	7.37
	Std. deviation	1.20	.46	1.53	.44	.52	.69
10	Mean	4.81	4.73	10.20	8.11	8.29	7.22
	Std. deviation	1.42	.93	1.40	.70	.90	.94

In the tabulated feedback points, the dyads’ wrong choice of adjectives (e.g., a big man vs. an old man), wrong active/passive voice (e.g., The car damaged.), and the inaccurate use/lack of punctuation were among the most frequent lower-level errors which received DIA. Likewise, the dyads’ weakness in writing clear and concise thesis statements was the most common higher-level error addressed by DIA.

To analyze the significance of the observed mean differences across the time factor (Task 1 to 10), five RM ANOVAs (corresponding to the number of higher-level and lower-level writing skills) were run (Table 4). In addition to the normality of the data reported in Table 2, the RM ANOVA assumes the homogeneity of covariance matrices, the assumption of sphericity, and the normality distribution of the residuals (errors). The results of the Box’s M statistics (M = 14.997, *p* = 0.950 > 0.01) indicated that the assumption of equivalence of covariance matrices was met. Mauchly's test of sphericity suggested that the assumption of sphericity was not violated, χ^2^(14) = 0.182, *p* = 0.927 > 0.01. Finally, the exploratory analysis of the homogeneity of residuals accounted for the normality of the data, *p* = 0.968 > 0.01.

**Table 4:** Repeated measures ANOVA on higher-level and lower-level writing skills

Component	Sum of squares	df	Mean square	F	Sig	Partial η^2^
*Organization*						
Between group	210.333	9	112.067	21.598	.072	.008
Within group	10.042	350	10.042	26.467	.059	.002
*Development*						
Between group	552.667	9	210.667	54.659	.089	.005
Within group	26.792	350	24.149	4.734	.090	.014
*Sentence structure*						
Between group	8070.167	9	6302.167	169.700	.000*	.190
Within group	53.133	350	26.567	2.154	.015*	.169
*Word choice & grammar*						
Between group	4056.000	9	2006.000	174.075	.000*	.212
Within group	48.875	350	9.775	2.015	.036*	.168
*Mechanics*						
Between group	4481.167	9	2538.167	114.037	.000*	.106
Within group	91.208	350	10.242	9.893	.006*	.182

*Significant at the .05 level (2-tailed)

The results of the RM ANOVAs in Table 4 indicate a significant progress of the dyads in the lower-level components of sentence structure (F (9, 350) = 169.700, *p* = 0.000, Partial η^2^ = 0.190), word choice & grammar (F (9, 350) = 174.075, *p* = 0.000, Partial η^2^ = 0.212), and mechanics of writing (F (9, 350) = 114.037, *p* = 0.000, Partial η^2^ = 0.106) with strong effect sizes, at the cost of no significant progress in the higher-level components of organization (F (9, 350) = 21.598, *p* = 0.072, Partial η^2^ = 0.008), and development (F (9, 350) = 54.659, *p* = 0.089, Partial η^2^ = 0.005) (for calculating effect size, cf. to Lenhard & Lenhard, 2016).

### Impact of DIA on sustainability of writing development

The second research question was examined by conducting descriptive statistical analysis and one-way ANOVA of the pretest, the immediate and delayed posttest gain scores.

As it can be seen in Table 5, the means of both immediate posttest (M = 25.10, SD = 1.514) and delayed posttest gain scores (M = 24.65, SD = 1.559) showed considerable improvement from the pretest (M = 14.19, SD = 1.650), with no considerable difference from each other. To explore the significance of this observable improvement, a one-way ANOVA was run. As Table 6 indicates, the mean differences across the three measurements were statistically significant, F (2, 105) = 388.003, *p* = 0.000, Partial η^2^ = 0.230, representing a strong effect size.

**Table 5:** Descriptive statistics: pretest, the immediate and delayed posttest scores

Test	N	Mean	Std. deviation	Std. error	95% confidence interval for mean	
					Lower bound	Upper bound
Pretest	36	14.19	1.650	.266	12.45	17.03
Immediate Posttest	36	25.11	1.514	.273	22.98	27.11
Delayed Posttest	36	24.65	1.559	.329	22.09	27.81

**Table 6:** Mean comparison: pretest, the immediate and delayed posttest scores

	Sum of squares	Df	Mean square	F	Sig. partial η2
Between groups	4143.629	2	2071.814	388.003	.000* .230
Within groups	560.666	105	5.339		
Total	4704.293	107			

According to the results of Tukey post-hoc comparison tests in Table 7,

1. The dyads significantly improved their writing performance from the pretest (M = 14.19) to the immediate posttest (M = 25.11) (Mean Difference = 13.220, *p* = 0.000).
2. The dyads also significantly improved their writing performance from the pretest (M = 14.19) to the delayed posttest (M = 24.65) (Mean Difference = 13.063, *p* = 0.000).
3. However, the dyads showed no significant improvement in their writing performance from the immediate posttest (M = 25.11) to the delayed posttest (M = 24.65) (Mean Difference = 0.170, *p* = 0.949).

**Table 7:** Post-hoc multiple comparison: pretest, the immediate and delayed posttest scores

(I)group	(J)group	Mean difference (I–J)	Std. error	Sig.	95% confidence interval	
					Lower bound	Upper bound
Pretest	Immediate posttest	13.220*	.380	.000*	12.980	14.980
	Delayed posttest	13.063*	.381	.000*	12.091	15.012
Immediate posttest	Pretest	13.220*	.380	.000*	12.980	14.980
	Delayed posttest	.170	.430	.949	− .670	.939

*Significant at the .05 level (2-tailed)

### Analysis of student reflective logs

To address the third research question which explored the potential impact of DIA on EFL learners’ engagement at behavioral, emotional, and cognitive modes, the content of the dyads’ responses to the pre-planned prompts were analyzed inductively. The content of the 10-week reflective logs was subjected to the theme frequency analysis (Emerson et al., 2011). The behavioral mode of engagement in collaborative writing was measured in terms of the overall length of time per task every dyad self-reported on writing and revising the weekly task. By comparison, the average time per task was similar across dyads and across tasks with an insignificant difference, M = 19.48 ± 3.38 min, Pearson χ^2^ (3960) = 0.70, *p* = 0.690, Cramer’s V = 0.10, interpreting a weak effect size.

The dyads’ responses to the second prompt were analyzed for their emotional mode of engagement in DIA. The three extracted themes were *a sense of social presence* (N = 79), *low anxiety* (N = 66), and *user-friendliness* of Google Meet (N = 47). To the majority of the dyads, receiving personalized, detailed DIA created a sense of community similar to a real classroom before the COVID-19 crisis. Accordingly, Dyad #3 on Task 5 was consent with the teacher’s monitoring of errors and online weekly presence, by stating that *“It is great [that] everything is watched [by the teacher] and we are not alone”*, or Dyad # 11 on Task 8 was satisfied with the localized focus of DIA and wrote that *“It is easy when the errors of our team are explained one by one every session”.* Also, they frequently referred to the stress-free environment of DIA in Google Meet as they could see their teacher and classmates every week. To Dyad # 7 on Task 4 wrote, *“When we see each other with our teacher who gives us feedback, it is so amazing! Just like the time before Covid!”.* Also, using Google Meet was addressed as a more convenient and easier platform than other CMC platforms. For instance, Dyad # 15 on Task 10 mentioned that *“Using features in Google Meet is much easier and faster than Skype”.*

The cognitive engagement in DIA was defined in terms of the dyads’ elaboration of their learning experience in every task completion. The three extracted themes were *the applicability and usefulness of DIA* (N = 113), *positive self-assessment of writing improvement* (N = 85), and *teacher linguistic bias or prioritization towards language form(s) over the content in DIA* (N = 23). The majority of dyads acknowledged the usefulness of DIA in completing writing tasks and revising them. For instance, Dyad # 6 expressed their positive impression of DIA on Task 4 by stating *“We should work harder because we wish to have good comments next session”*, or Dyad # 8 on Task 10 mentioned that *“We learned a lot about different forms of verbs because our errors were explained every session. Not only a general explanation but our errors!”*. They also frequently appreciated DIA as a source of motivation to write more and to have observable progress. In doing so, Dyad # 7 on Task 8 emphasized a fast improvement in their writing by stating, *“This is like a private classroom that the teacher gives all her attention to us!”*

The participants were not always positive about DIA in their reflective logs. Some dyads were critical of ‘*a sort of sensitivity’* or prioritization in teacher DIA to language forms, such as grammaticality, choice of words, or mechanics of writing. For instance, Dyad # 2 on Task 5 criticized the linguistic bias and wrote, *“The teacher wished to see big adjectives all the time! I don’t know many synonyms”*, or Dyad # 7 showed their disappointment on Task 9 by stating “*We were waiting for an Excellent on our report, but our misspellings were more important!”*

## Discussion

The discussion of the first research question is two-fold. The impact of DIA on the participants’ progress in collaborative writing can be argued from the viewpoint of Vygotsky’s social constructivism (1987). As a type of cognitive feedback, DIA is a learner-centered, process-oriented approach to language assessment that mediates the L2 learners to co-construct feedback literacy in collaboration (Sutton, 2012). In other words, through the teacher's ongoing observation, detailed error description, and student–student interactions in the course of collaborative writing, the peers are collectively scaffolded and reach their *zone of proximal development* (Vygotsky, 1987). Embodied in the DIA framework, the dyads in the study could systematically use their writing output as their learning input, and actively engage in their writing and revising process. Moreover, the DIA provided the participants with adequate explicit knowledge *to acquire* the target language form and *to self-monitor* their writing performance.

On the other hand, the research findings indicated that unlike the lower-level skills of writing which were significantly improved by the teacher's DIA, the higher-level skills remained unaffected. This finding can be argued from the cognitive psychology perspective, which underlies the cognitive processes the students undertake to analyze the received DIA. Accordingly, *making sense* of the feedback is dependent on the content of the task and is central to *how well* the recipients of the feedback can use it in subsequent writing tasks (Link et al., 2020). As Alderson stated, “the essence of a diagnostic test must be to provide meaningful information to users which they can understand and upon which they or their teachers can act” (2005, p. 208). Therefore, due to the complexity of the higher-level qualities, such as text organization or developing academic genre of writing, the students most likely failed to comprehend the received DIA (Li, 2010). As a result, the teacher’s regular DIA seemed to promote writing at words and sentence level with no substantial improvement at pragmatic, or discursive planes. Considering the intermediate language proficiency profile of the participants, this unequal achievement of higher-level and lower-level skills might be justified by Seliker’s Interlanguage approach (1972) to language development. Accordingly, independently from the timeliness, amount, and types of feedback the L2 learners receive from the teacher and peers, their linguistic patterns should gradually evolve as time goes by, following a pre-determined natural order. Moreover, from Krashen’s (1982) cognitive psychology perspective, learning the complexities in an L2 is a self-paced, gradual cognitive process that can not be bypassed by intervening feedback. This notion, however, does not challenge the importance of using differentiated strategies to provide various feedback types including DIA.

Among a few experimental studies on the DIA in the SLA literature, Tozcu (2016) was the only one concerned with the pedagogical role of DIA in improving language skills which came up with contradictory results to those in this study. Tozcu investigated the impact of DIA with 24 military students learning Turkish in a full-time military language program in the United States. In a pretest–posttest design, the participants’ performance on oral narrative tasks was subjected to DIA in the treatment group, while the participants in the control group received only post-narration reading comprehension questions. Tozcu (2016) reported a significant improvement in the accuracy of basic sentence structures as well as discursive cohesive devices by the treatment group. In other words, DIA could positively impact both the lower-level and higher-level oral skills of the participants. One possible argument for the findings in this study is the high level of task engagement by the military participants and the intensity of the language course they attended. However, from the research methodology perspective, the researcher’s conclusion is open to an argument due to the small size of the participants in each group.

The discussion of the second research question which investigated the sustainability of the DIA impact on writing improvement is anchored in the important feature of DIA as a type of cognitive feedback (Jang & Wagner, 2014). Unlike evaluative and corrective feedback which focus mostly on the immediate task outcome, cognitive feedback provides descriptive information about the traces of cognitive processes the L2 learners use to “*restructure* their prior knowledge incompatible with future learning tasks” (Jang & Wagner, 2014, p. 4). Therefore, as a type of cognitive feedback, DIA can result in L2 learners’ self-regulated learning and long-term monitoring of the gap between their current state of knowledge and the target learning goal. The insignificant difference between the participants’ performance on the immediate and delayed posttests after a four-week non-interventional interval can reassure the sustainability and the constructive role of DIA in improving writing task performance.

According to Lee (2015), this notion supports the consequential validity of DIA, as its positive impacts might predict sustainable L2 learning. DIA is even more salient to Iranian EFL learners due to its historical relevance. In other words, because Iranian EFL learners have received a substantial amount of formal English grammar instructions at school, their uptake of DIA is deemed to be sustainable (Hashemian & Farhang-Ju, 2018). The growing L2 research literature on the effectiveness of DIA (Kazemi & Tavassoli, 2020; Lee, 2015; Nikmard & Tavassoli, 2020; Pourdana & Rad, 2017; Rafi et al., 2022; Saadatmandi et al., 2018) supports the findings on the second research question but mostly in reading and listening improvement.

The discussion of the third research question is secured in the critical concepts of feedback literacy and reflective thinking as two major by-products of DIA. Through regular teacher DIA, the dyads could feel confident about their understanding of feedback and develop their capacity to translate the impact of DIA into action (Sutton, 2012). The subsequent student reflective logs could enhance the L2 learners’ monitoring of their behaviors, emotions, and work-in-progress. The findings on the third research question indicated active behavioral engagement of the participants in extending their time to write and revise. Such a dynamic self-regulated learning behavior has been supported as the action-based impact of DIA by several researchers (Carless & Boud, 2018; Doe, 2015; Mahdi & Al Khateeb, 2019). Regarding the emotional engagement of participants in DIA, the students experienced a low-stress environment, community membership, and simplicity in computer-assisted DIA on Google Meet. The findings corroborate those in Rafi et al. (2022) and Trowler (2010) who reported the impacts of DIA on the students’ confidence, motivation, and positive learning attitude.

Contrary to the findings in this study, a few studies provided evidence for DIA being blamed by the low-proficiency L2 learners as a source of embarrassment and apprehension (Jang & Wagner, 2014; Kunnan & Jang, 2009). The participants’ cognitive engagement was depicted by their approval of the accelerating role of DIA in their L2 learning progress. Despite insisting on the positive role of DIA, the participants also brought up the issue of the teacher's linguistic bias towards language forms which could be interpreted as “the mismatch between teachers’ and students’ expectations” (Chong, 2020, p.1), and the common reaction of the students to the teacher corrective feedback (Pourdana et al., 2021). The findings were also in line with several qualitative research studies which reported students’ low feedback literacy as the potential source of misinterpreting the teacher formative assessment (Carless & Boud, 2018; Price et al., 2010).

In this study, the DIA practice was carried out on the CMC platform of Google Meet to surpass the restrictions imposed by the COVID-19 pandemic. Fortunately, the computer-assisted DIA could turn into a great opportunity to eliminate the challenges of time and space in the traditional face-to-face classroom environment. Therefore, the facilitative role of Google Meet as a *forum* to implement the teacher’s DIA practice and learners’ social interactions was effectively exploited. Yet, to carry out the DIA practice on L2 writing and speaking, the remarkable progress in the web-based automated feedback technology can serve as a second-rater to supplement the human rater’s score. The effectiveness of online interactive applications such as Google Meet in L2 educational contexts has been reported in several experimental studies and promoted for setting a relaxed and low-stress learning environment (Rafi et al., 2022), enhancing active teamwork (Flores, 2020), and acquiring social skills (Murphy, 2020). Moreover, computer-based diagnostic tests like DELTA and DIALANG offer generic diagnostic feedback as to what learners might do to improve in particular areas (Harding et al., 2015).

## Conclusion

This study provided evidence for the impact of computer-assisted DIA on EFL learners’ collaborative writing who significantly improved the lower-level writing skills in their subsequent drafts with no sign of significant improvement in the higher-level writing skills. Moreover, the DIA was reported to have both short-term and sustainable impacts on EFL learners’ writing achievement which were echoed in their immediate and delayed post-intervention summative assessment. Finally, DIA on Google Meet online platform could actively engage the students in participatory behaviors, so that they spent a large amount of time on collaborative writing (i.e., behavioral engagement). They also found the computer-assisted DIA highly practical, stress-free, and user-friendly (i.e., emotional engagement). Finally, the participants had a positive observation of their writing improvement, although they had some objections to the teacher’s unequal focus on language form and content (i.e., cognitive engagement).

The current study suggests that the EFL learners can largely benefit from the DIA that regularly and systematically targets their myriad of needs, goals, strengths, and weaknesses. However, the language assessment literature has offered very little guidance on how DIA might effectively be conducted and how its use might be validated. In other words, DIA has been ignored and rarely problematized in the L2 literature. Calling for further research in this venue, Alderson (2005) argued that “relatively little has been written about diagnostic testing in second and foreign language learning… and there is a degree of confusion in the literature about what exactly diagnostic tests are and how they should be constructed” (p. 13). Therefore, the research on language assessment needs a change of direction to become more diagnostic in practice. By the same token, DIA should become an integral part of the language curriculum, language teacher professional development programs, and L2 teaching practice.

The arguments in this research are still inconclusive due to some important ecological and methodological limitations. One of the major restrictions imposed on this study was the COVID-19 pandemic which caused countless limitations to select the participants, the data collection, log analysis, and regular discussions. From the research methodological perspective, the author selected the research participants non-randomly among the volunteers with a high level of enthusiasm, task engagement, and computer literacy. Also, the author did not intend to isolate the effects of collaboration from DIA by including a comparison group in this study. However, as it was meticulously mentioned by an anonymous reviewer of this paper, the collaboration of the dyads on the 10-week writing tasks could have a confounding impact on their solo performance on the immediate and delayed posttests which should be considered a limit to the generalizability of the findings.

## Data Availability

Contact the author for data requests.

## Appendix 2

A Sample of Data Commentary Task

People with different blood types respond to the COVID-19 infection differently. This bar graph illustrates the susceptibility of ABO blood types to the COVID-19 infection. In 300 words, write down your report on this bar graph.

## Abbreviations

AfL: Assessment for learning
DIA: Diagnostic assessment
CMC: Computer-mediated communication
ICT: Information and communication technologies
RM ANOVA: Repeated measures analysis of variance
L2: Second/Foreign language
TEFL: Teaching English as a Foreign Language
WVDE: West Virginia Department of Education

## References

[CR1] Alderson, J. C. (2005). *Diagnosing foreign language proficiency: The interface between learning and assessment.* Continuum.

[CR2] Alderson JC, Brunfaut T, Harding L (2015). Towards a theory of diagnosis in the second and foreign language assessment: Insights from professional practice across diverse fields. Applied Linguistics.

[CR3] Al-Maroof RS, Salloum SA, Hassanien AE, Shaalan K (2020). Fear from COVID-19 and technology adoption: The impact of Google Meet during Coronavirus pandemic. Interactive Learning Environments.

[CR4] Baber H (2020). Determinants of students’ perceived learning outcome and satisfaction in online learning during the pandemic of COVID-19. Journal of Education and E-Learning Research.

[CR5] Behbahani SMK, Pourdana N, Maleki M, Javanbakht Z (2011). EFL task-induced involvement and incidental vocabulary learning: Succeeded or surrounded. IPEDR Proceeding of International Conference on Languages, Literature, and Linguistics, Singapour.

[CR6] Best JW, Kahn JV (2006). Research in education.

[CR7] Bonner E, Garvey K, Miner M, Godin S, Reinders H (2022). Measuring real-time learner engagement in the Japanese EFL classroom. Innovation in Language Learning.

[CR8] Butler DL, Winne PH (1995). Feedback and Self-Regulated Learning: A Theoretical Synthesis. Review of Educational Research.

[CR9] Byrne BM (2010). Structural equation modeling with AMOS: Basic concepts, applications, and programming.

[CR10] Cárcamo B (2020). Classifying written corrective feedback for research and educational purposes: A typology proposal. Profile: Issues in Teachers’ Professional Development.

[CR11] Carless D, Boud D (2018). The development of student feedback literacy: Enabling uptake of feedback. Assessment & Evaluation in Higher Education.

[CR12] Chong SW (2020). Reconsidering student feedback literacy from an ecological perspective. Assessment & Evaluation in Higher Education.

[CR13] Csapó, B., & Molnár, G. (2019). Online diagnostic assessment in support of personalized teaching and learning: The eDia system. *Frontiers in Psycholology, 10*(1522). 10.3389/fpsyg.2019.01522 10.3389/fpsyg.2019.01522 PMC6617473 31333546

[CR14] Cumming A (2001). Learning to write in a second language: Two decades of research. International Journal of English Studies.

[CR15] Cumming A (2015). Design in four diagnostic language assessments. Language Testing.

[CR16] Diagnosis. (2013). In *The Merriam-Webster online dictionary*. Retrieved March 9, 2022, from www.merriam-webster.com/dictionary/diagnosis.

[CR17] Doe C (2015). Student interpretations of diagnostic feedback. Language Assessment Quarterly.

[CR18] Emerson RM, Fretz RI, Shaw LL (2011). Writing ethnographic fieldnotes.

[CR19] Flores MA (2020). Preparing teachers to teach in complex settings: Opportunities for professional learning and development. European Journal of Teacher Education.

[CR20] Fredricks JA, Blumenfeld PC, Paris AH (2004). School engagement: Potential of the concept, state of the evidence. Review of Educational Research.

[CR21] Fredricks JA, Eccles JS (2002). Children's competence and value beliefs from childhood through adolescence: Growth trajectories in two male-sex-typed domains. Developmental Psychology.

[CR22] Gorin, J. S. (2007). Test Construction and diagnostic testing. In J. Leighton & M. Gierl (Eds.), *Cognitive diagnostic assessment for education: Theory and applications*, (pp. 173–201). Cambridge University Press.

[CR23] Harding, L., Alderson, C. J., & Brunfaut, T. (2015). Diagnostic assessment of reading and listening in a second or foreign language: Elaborating on diagnostic principles. *Language Testing,* 1–20.

[CR24] Hashemian M, Farhang-Ju M (2018). Effects of metalinguistic feedback on grammatical accuracy of Iranian field (in)dependent L2 learners' writing ability. Journal of Research in Applied Linguistics.

[CR25] Hismanoglu M, Hismanoglu S (2011). Task-based language teaching: What every EFL teacher should do. Procedia-Social and Behavioral Sciences.

[CR26] Jang EE, Wagner M, Kunnan AJ (2014). Diagnostic feedback in the classroom. The companion to language assessment, Volume II: Approaches and development.

[CR27] Kazemi, N., & Tavassoli, K. (2020). The comparative effect of dynamic vs. diagnostic assessment on EFL learner’s speaking ability. *Research in English Language Pedagogy, 8*(2), 223–241.

[CR28] Krashen SD (1982). Principles and practice in second language acquisition.

[CR29] Kunnan A, Jang EE, Long M, Doughty C (2009). Diagnostic feedback in language testing. The handbook of language teaching.

[CR30] Lam R, Burns A, Siegel J (2018). Promoting self-reflection in writing: A showcase portfolio approach. International perspectives on teaching skills in ELT.

[CR31] Lee YW (2015). Diagnosing diagnostic language assessment. Language Testing.

[CR32] Lei M, Medwell J (2021). Impact of the COVID-19 pandemic on student teachers: How the shift to online collaborative learning affects student teachers’ learning and future teaching in a Chinese context. Asia Pacific Education Review.

[CR33] Lenhard, W., & Lenhard, A. (2016). *Calculation of effect sizes.* Psyhometrica, Bibergau.

[CR34] Li S (2010). The effectiveness of corrective feedback in SLA: A meta-analysis. Language Learning.

[CR35] Link S, Mehrzad M, Rahimi M (2020). Impact of automated writing evaluation on teacher feedback, student revision, and writing improvement. Computer Assisted Language Learning.

[CR36] Mahdi HS, Al Khateeb AA (2019). The effectiveness of computer-assisted pronunciation training: A meta-analysis. Review of Education.

[CR37] Mahmoudi F, Buğra C (2020). The effects of using rubrics and face to face feedback in teaching writing skill in higher education. International Online Journal of Education and Teaching (IOJET).

[CR38] Martin, D., & Miller, C. (2003). *Speech and language difficulties in the classroom.* David Fulton.

[CR39] Murphy MPA (2020). COVID-19 and emergency eLearning: Consequences of the securitization of higher education for post-pandemic pedagogy. Contemporary Security Policy.

[CR40] NBCT Office of Assessment West Virginia Department of Education. (2015). Artificial intelligence scoring of student essays: West Virginia’s experience.

[CR41] Nikmard F, Tavassoli K (2020). The effect of diagnostic assessment on EFL learners’ performance on selective and productive reading tasks. Journal of Modern Research in English Language Studies.

[CR42] Pourdana N, Bornaki F, Moayedi Fard Z, Sarkhosh SZ (2012). Test-taking strategies and performance on reading comprehension tests by Iranian EFL learners. International Journal of Applied Linguistics & English Literature.

[CR43] Pourdana N, Nour P, Yousefi F (2021). Investigating metalinguistic written corrective feedback focused on EFL learners’ discourse markers accuracy in mobile-mediated context. Asian-Pacific Journal of Second and Foreign Language Education.

[CR44] Pourdana N, Rad MS (2017). Differentiated instructions: Implementing tiered listening tasks in mixed-ability EFL context. Journal of Modern Research in English Language Studies.

[CR45] Pourdana N, Sahebalzamani S, Rajeski JS (2014). Metaphorical awareness: A new horizon in vocabulary retention by Asian EFL learners. International Journal of Applied Linguistics and English Literature.

[CR46] Price M, Handley K, Millar J, O’Donovan B (2010). Feedback: All that effort, but what is the effect?. Assessment & Evaluation in Higher Education.

[CR47] Purwanto E, Tannady H (2020). The factors affecting intention to use Google Meet amid online meeting platforms competition in Indonesia. Technology Reports of Kansai University.

[CR48] Putra RWP (2021). Improving the students’ motivation in learning English through Google Meet during online learning. English Learning Innovation.

[CR49] Rafi, F., Pourdana, N., & Ghaemi, F. (2022). Computer-mediated diagnostic assessment of mixed-ability EFL learners’ performance on tiered tasks: Differentiating mediation on Google Meet™. *Journal of Modern Research in English Language Studies.*10.30479/jmrels.2021.16118.1950

[CR50] Ritter, O. N. (2018). *Integration of educational technology for the purposes of differentiated instruction in secondary STEM education*. Unpublished doctoral dissertation. The University of Tennessee.

[CR51] Saadatmandi M, Khiabani SM, Pourdana N (2018). Teaching English pragmatic features in EFL context: A focus on request speech acts. Theory and Practice in Language Studies.

[CR52] Selinker L (1972). Interlanguage. International Review of Applied Linguistics in Language Teaching.

[CR53] Sette-de-Souza PH (2020). Motivating learners in the pandemic period through WhatsApp and Google Meet. Journal of Dental Education.

[CR54] Storch N (2013). Collaborative writing in L2 classrooms.

[CR55] Sutton P (2012). Conceptualizing feedback literacy: Knowing, being, and acting. Innovations in Education and Teaching International.

[CR56] Tomlinson CA (2014). The differentiated classroom: Responding to the needs of all learners.

[CR57] Tozcu A (2016). The effectiveness of diagnostic assessment on the development of Turkish language learners’ narrative skills as an oral proficiency interview (OPI) task. Journal of the National Council of Less Commonly Taught Languages.

[CR58] Trowler, V. (2010). *Students engagement literature review.* The Higher Education Academy.

[CR59] Vygotsky, L. S. (1987). Thinking and speech. In R. W. Rieber, & A. S. Carton (Eds.), *The collected works of L. S. Vygotsky: Vol. 1: Problems of general psychology* (pp. 239–285). Plenum.

[CR60] Weimer, M. (2002). *Learner-centered teaching: Five key changes to practice*. Wiley.

[CR61] Wigglesworth G, Storch N (2009). Pairs versus individual writing: Effects on fluency, complexity, and accuracy. Language Testing.

[CR62] Yin M, Sims J, Cothran D (2012). Scratching where they itch: Evaluation of feedback on a diagnostic English grammar test for Taiwanese university students. Language Assessment Quarterly.

